# Protective Role of Phenolic Compounds from Whole Cardamom (*Elettaria cardamomum* (L.) Maton) against LPS-Induced Inflammation in Colon and Macrophage Cells

**DOI:** 10.3390/nu15132965

**Published:** 2023-06-29

**Authors:** Shareena Sreedharan, Vimal Nair, Luis Cisneros-Zevallos

**Affiliations:** 1 Department of Horticultural Sciences, Texas A&M University, College Station, TX 77843-2133, USA; 2 Department of Food Science and Technology, Texas A&M University, College Station, TX 77843-2133, USA

**Keywords:** cardamom, *Elettaria cardamomum* (L.) Maton, inflammation, polyphenols, oxidative stress, pro-inflammatory genes and nuclear receptors, mode of action, colon and macrophage cells

## Abstract

The chemical profiling of phenolic and terpenoid compounds in whole cardamom, skin, and seeds (*Elettaria cardamomum* (L.) Maton) showed 11 phenolics and 16 terpenoids, many of which are reported for the first time. Herein, we report the anti-inflammatory properties of a methanolic extract of whole cardamom in colon and macrophage cells stimulated with an inflammatory bacteria lipopolysaccharide (LPS). The results show that cardamom extracts lowered the expression of pro-inflammatory genes *NFkβ*, *TNFα*, *IL-6*, and *COX2* in colon cells by reducing reactive oxygen species (ROS) while not affecting *LXRα*. In macrophages, cardamom extracts lowered the expression of pro-inflammatory genes *NFkβ*, *TNFα*, *IL-6*, and *COX2* and decreased NO levels through a reduction in ROS and enhanced gene expression of nuclear receptors *LXRα* and *PPARγ.* The cardamom extracts in a range of 200–800 μg/mL did not show toxicity effects in colon or macrophage cells. The whole-cardamom methanolic extracts contained high levels of phenolics compounds (e.g., protocatechuic acid, caffeic acid, syringic acid, and 5-O-caffeoylquinic acid, among others) and are likely responsible for the anti-inflammatory and multifunctional effects observed in this study. The generated information suggests that cardamom may play a protective role against low-grade inflammation that can be the basis of future in vivo studies using mice models of inflammation and associated chronic diseases.

## 1. Introduction

*Elettaria cardamomum* (L.) Maton is a plant native to India. However, in accordance with demand and economic interest, this spice is also cultivated in Guatemala, Ski Lanka, Nepal, Vietnam, Laos, and El Salvador. Guatemala is the largest cardamom producer in the world with ~35,000 yearly metric tons and with 70% of its production involving 350,000 small farmers. However, low prices and fluctuations in cardamom market demands generate a negative economic and social impact on small farmers and their families [1], and may affect the ability to produce, maintain life quality, and even decisions to immigrate. One alternative to increase demand and open new markets for crops is to explore their nutraceutical properties targeting high-value health markets such as functional foods and dietary supplements [2]. For instance, cardamom has been shown to have potential anti-inflammatory effects [3,4,5,6,7,8,9,10,11], exert enzyme inhibition [12] and control blood pressure [13]. Certain active compounds present in cardamom such as 1,8-cineole, terpinyl acetate, limonene, terpinolene, and myrcene reduced vascular risk [14] and improved glucose tolerance and oxidative stress in the liver when tested in diet-induced obese rats [15] and have shown improvement in non-alcoholic hepatic steatosis [16]. Chronic diseases in society have been closely associated to low-grade and chronic inflammation, and preventive and therapeutic strategies have been proposed by using nutraceuticals, dietary supplements, and foods [2]. 

Low-grade inflammation may be triggered by microbial dysbiosis induced by diet as well as by an initial state of obesity among other factors causing chronic diseases, such as metabolic syndrome. Under an inflammation state, undesirable disease-causing adipocytokines—such as TNF-α, causing insulin resistance, and PAI-I, associated with clot formation—may be exacerbated in the adipose tissue as macrophages invade them, producing even more TNF-α, MCP-1, IL-6, and NO in a feedback loop and causing a state of chronic inflammatory reactions (e.g., activation of inflammatory signaling molecules such as JNK, NFK-β, and others), resulting in further exacerbation of the metabolic syndrome. Many of these circulating chemicals affect the vascular endothelial tissue as well, causing inflammation that can be exacerbated by LDL oxidation and infiltration as well as initiating atherosclerosis and thrombosis mediated by platelet aggregation [17]. 

Targeting disease-related inflammatory components under this complex scenario could be a useful strategy for the prevention and amelioration of obesity-related pathologies, including insulin resistance and atherosclerosis as well as other chronic diseases. 

Herein, we hypothesize that cardamom bioactive compounds may have multiple functions and work against inflammation in different fronts simultaneously, either by reducing inflammation in different types of cells and exerting their mode of action through the suppression of reactive oxygen species and/or by regulating the expression of nuclear receptors, which modulate the pro-inflammatory transcription factor NFK-β. In the present study, we characterized cardamom bioactives and determined their mode of action against inflammation in colon and macrophage cells. The results of our investigation will benefit the cardamom industry by potentially increasing the value, marketability, and profitability of cardamom.

## 2. Materials and Methods

### 2.1. Chemicals and Reagents

Diphenyleneiodonium (DPI), 2′,7′-dichlorofluorescin diacetate (DCFA), lipopolysaccharide (LPS), sodium nitrite solution, Griess reagent, Dulbecco’s Modified Eagle Medium (DMEM)/low glucose, phenol red-free DMEM/low glucose, penicillin/streptomycin mixture, DMSO, and fetal bovine serum (FBS) were purchased from Sigma (St. Louis, MO, USA). Glucose was purchased from Acros Organics (Fair Lawn, NJ, USA), and sodium bicarbonate was purchased from Mallinckrodt Chemicals (Phillipsburg, NJ, USA). Caco-2 cells and RAW 264.7 macrophages (cell line TIB-71™) were acquired from the American Type Culture Collection (ATCC) (Manassas, VA, USA).

### 2.2. Cardamom Samples

Cardamom parchment or pods from the northern zone of Coban (Nimlajacoc) in Guatemala was used in this study. The samples of cardamom parchment (whole cardamom) were separated into two parts, the outer shell, or skin, and the “gold samples” that are the inner seeds. Non-volatile polyphenols were obtained by a methanolic extraction, and the profiles was evaluated by liquid chromatography coupled to mass spectrometry (LC-MS). Volatile terpenoids were obtained by a steam distillation method to extract the essential oils of cardamom while the profiles were determined by gas chromatography coupled to mass spectrometry (GC-MS). In general, ~175.8 ± 5.6 g of whole cardamom yields 119 ± 5 g of seeds and 56.8 ± 1.4 g of skin, representing seeds and skins as ~67.6% and 32.3% by weight (*n* = 100), respectively.

### 2.3. Sample Preparation for Polyphenol Analysis and LC-MS Profiling

Grounded powders of ~1 g cardamom seed, ~1 g cardamom skin, and ~0.6 g of whole cardamom were each dissolved in 10 mL methanol and stirred for 15 h at 4 °C. The methanolic extracts were then centrifuged and concentrated at 4000 rpm (2147× *g*) at 45 °C until all of the volatile solvents were evaporated in a Centrivap concentrator connected to a cold trap (Labconco, Kansas City, MO, USA). The dry extract yields were ~126.2, 80.4, and 85.2 mg for seed, skin, and whole cardamom, respectively. 

The extracts obtained from seed, skin, and whole cardamom were re-dissolved in 10 mL methanol and the appropriate amount of extract was taken (as given above) to make a concentration of ~5 mg/mL for each sample and filtered with a 0.22 µm PTFE filter, and a volume of 10 µL was injected into the LC-MS in 3 replicates. 

Individual phenolic compounds were determined on a Surveyor HPLC/MS system equipped with a Surveyor 2000 quaternary pump, a Surveyor UV 2000 PDA detector, and an autosampler using a C_18_ reverse-phase (150 mm × 4.6 mm, Atlantis, Waters, Wexford, Ireland; particle size = 5 μm) column. The system was connected to a LCQ Deca XP Max MSn system (Thermo Finnigan, San Jose, CA, USA) with a Z-spray ESI source run by Xcalibur software, version 1.3 (Thermo Finnigan-Surveyor, San Jose, CA, USA). A flow rate of 0.25 mL/min was used for the mobile phase, while the elution gradients were performed with solvent A, consisting of acetonitrile/methanol (1:1) (containing 0.5% formic acid); and solvent B, consisting of water (containing 0.5% formic acid). The elution conditions applied to the starting condition were 0–2 min, 2% A, 98% B; 3–5 min, 5% A, 95% B; 5–7 min, 25% A, 75% B; 7–12 min, 55% A, 45% B; 12–24 min 55% A–80% A, 24–27 min held isocratic at 80% A, 28–30 min 90% A, 10% B; 31–33 min held isocratic, 100% A; and 34–40 min, 2% A, 98% B. The chromatograms were monitored at 280 nm, and complete spectral data were recorded in the range 200–600 nm. ESI was performed in the negative ionization mode, nitrogen was used as sheath gas with a flow of 59 arbitrary units, and He gas was used as dampening gas. The capillary voltage was −4.17 V; spray voltage, 5 kV; capillary temperature, 275 °C; and tube lens voltage, −55 V. Collision energies of 30% were used for the MSn analysis. The quantification of individual phenolics was determined by using peak chromatograph areas and a chlorogenic acid standard curve. It was reported as mg of chlorogenic acid equivalents/100 g, and their sum was reported as total phenolics.

### 2.4. Sample Preparation for Terpenoid Analysis and GC-MS Profiling

Essential oils were extracted by taking ~5 g of each sample from seed, skin, and whole cardamom then added to 500 mL of distilled water and subjected to steam distillation to obtain the essential oils. The oil obtained was dried over anhydrous sodium sulphate and stored in amber and air-tight sealed vials at 0 °C until analysis and testing. Essential oil yields were ~20.2, 21.1, and 20.8 mg for seed, skin, and whole cardamom, respectively.

GC-FID analyses were carried out on TRACE GC Ultra gas chromatograph equipped with an auto sampler with a RX-5MS capillary column (60 m × 0.25 mm, 0.25 μm film thickness) and it was coupled to a mass spectrometer (Thermo Scientific, WestPalm, FL, USA). The column temperature was programmed to rise from 50 to 280 °C at a rate of 3 °C/min. A flow rate of 1.5 mL/min was used for the carrier gas helium. The scan time and mass range were 1 s and 50–550 *m*/*z*, respectively, and the acquired data were processed using an Xcalibur software (Thermo Fisher Scientific, version 2.0.7). The peaks obtained were tentatively identified by comparing their mass spectra with those included in the NIST-05a. The essential oils were injected at a concentration of 2.0 mg/mL for cardamom seed, skin, and whole cardamom.

### 2.5. Cell Culture for Inflammation Studies

Raw 264.7 macrophages were grown in the DMEM–low glucose (pH 7.2–7.4) containing 4 g/L glucose, 3.7 g/L sodium bicarbonate, 10% fetal bovine serum (FBS), and antibiotics (100 units/mL penicillin and 100 μg/mL streptomycin) in a humidified atmosphere with 5% CO_2_ at 37 °C. Cells from passages 3 to 11 were used in this study. The cells were plated at 0.5 × 10^4^ cells/well in 96-well black- or clear-bottom plates (Costar, Cambridge, MA, USA) for MTS test, NO/ROS, and at 0.5 × 10^6^ cells/well in 6-well plates (BD Biosciences, Franklin Lakes, NJ, USA), for gene expression studies. The cells were pretreated 5 h with whole-cardamom extract and afterwards treated with the growth medium containing 1 µg/mL LPS for 19 h, co-incubating with whole-cardamon extracts (total 24 h). 

The Caco-2 cells from human colon adenocarcinoma were used between passages 5 and 20 and cultured in high-glucose DMEM (pH 7.2–7.4) medium supplemented with 4 g/L glucose, 3.7 g/L sodium bicarbonate, 20% fetal bovine serum (FBS), and antibiotics (100 units/mL penicillin and 100 μg/mL streptomycin) in a humidified atmosphere with 5% CO_2_ at 37 °C. For MTS, ROS, and NO assays, cells were seeded at a density of around 8  ×  10^3^ cells/well in 96-well black- or clear-bottom plates (Costar, Cambridge, MA, USA) and at 0.5 × 10^6^ cells/well in 6-well plates (BD Biosciences, Franklin Lakes, NJ, USA) for gene expression studies. Cells were pretreated 5 h with whole-cardamom extract and afterwards treated with the growth medium containing 50 µg/mL LPS for 19 h, co-incubating with whole-cardamom extracts (total 24 h). Whole-cardamom extracts were dissolved in the growth medium containing 0.5% DMSO, and media containing DMSO were used as a control in all experiments.

### 2.6. Cell Viability Test

Cytotoxicity of cardamon extract on Raw264.7/Caco-2 cells was studied using the MTS assay (Promega Corp., Madison, WI, USA), according to the manufacturer’s instructions. Briefly, Raw264.7 and Caco-2 cells were plated at a density of 0.5 × 10^4^ and 8  ×  10^3^ cells/well, respectively, in 96-well plates (Costar, Cambridge, MA, USA) and incubated at 37  °C, 5% CO_2_ with DMEM medium for 24 h. After 24 h, the medium was removed and a fresh medium supplemented with different concentrations (200–1000 µg/mL) of cardamom extract was added for 24 h. A 20 µL MTT solution was then added to the plate and incubated at 37 °C for 3 h. The amount of formazan product formed in the MTT assay that is directly proportional to the number of living cells in the culture was measured at 490 nm.

### 2.7. Detection of Extracellular Nitric Oxide and Intracellular Reactive Oxygen Species Production

Cells were plated at 0.5 × 10^4^ (Raw 264.7) and 8 × 10^3^ (Caco2) cells/well in 96-well black- and clear-bottom plates (Costar, Cambridge, MA, USA) for ROS and NO assays, respectively, and cultured overnight. The cells were treated with cardamom extract for 5 h followed by stimulation with LPS (50 µg/mL for Caco2 and 1 µg/mL for Raw 264.7) for 19 h, and they were co-incubated with the cardamom extract. Thereafter, the cells and the medium were used for the ROS and NO detections, respectively. 

The nitric oxide (NO) production was assessed as the accumulation of nitrite (NO_2_^−^) in the medium using a colorimetric reaction with Griess reagent. Briefly, 50 µL of the cell culture supernatants obtained 24 h after the LPS treatment and 100 mM sodium nitrite solution was diluted with nanopure water for the preparation of standards from 10 to 100 µM. Afterwards, the cell supernatants and standards were mixed with an equal (1:1) volume of Griess reagent, and absorbance was measured at 540 nm using a 96-well microplate reader (Synergy HT, Bio-Tek Instruments, Inc., Winooski, VT, USA). The intracellular ROS production was measured by 2′,7′-dichlorofluorescin diacetate (DCFA). Briefly, the cell culture medium was removed by aspiration 24 h after the LPS challenge, and subsequently, cells were exposed to 10 µM DCFA in phenol red/FBS-free DMEM for 30 min then washed twice with the phenol red/FBS-free DMEM. Afterwards, fluorescence was read immediately at wavelengths of 485 nm for excitation and 528 nm for emission on a 96-well microplate reader (Synergy HT, Bio-Tek Instruments, Inc., Winooski, VT, USA).

### 2.8. Total RNA and Gene Expression Analysis (Real-Time qRT-PCR)

After LPS treatment for 19 h, total RNA was extracted from Caco2 and Raw 264.7 cells using the TRIzol^®^ Reagent (Invitrogen, Carlsbad, CA, USA), according to the manufacturer’s instructions. A NanoDrop ND-1000 spectrophotometer (NanoDrop Technologies, Willmington, DE, USA) was used to measure RNA concentration. A total of 1 µg RNA, treated with DNase I to avoid DNA contamination, was reverse-transcribed into cDNA using the SuperScript III First-Strand Synthesis SuperMix (Invitrogen, Carlsbad, CA, USA), following the manufacturer’s protocol. Thereafter, the cDNAs were used for real-time qRT-PCR analyses using Power SYBR Green PCR Master Mix (Applied Biosystems, Foster City, CA, USA), following the manufacturer’s instructions. A 7900 HT Sequence Detection System (Applied Biosystems, Foster City, CA, USA) was used for cDNA amplification. Finally, the relative expression of each gene was normalized by β-actin [18,19] and was calculated following the comparative Ct method (ΔΔCt), also known as the 2^−ΔΔCt^ method [20]. Gene expression assays were performed in triplicate with three readings per replicate. The primer sets for the target gene expressions are listed in Table 1 and were provided by Integrated DNA Technologies (IDT, Coralville, IA, USA).

### 2.9. Statistical Analysis

Assays were performed in triplicate for each sample and reported as average values + SD. A one-way analysis of variance (ANOVA) was used to analyze the data, and mean separation was performed by the Tukey test at a 5% error using the JMP pro v10.0 software.

## 3. Results and Discussion

### 3.1. Polyphenol Profiles from Whole Cardamom, Skin, and Seeds by LC-MS

**Figure 1:**
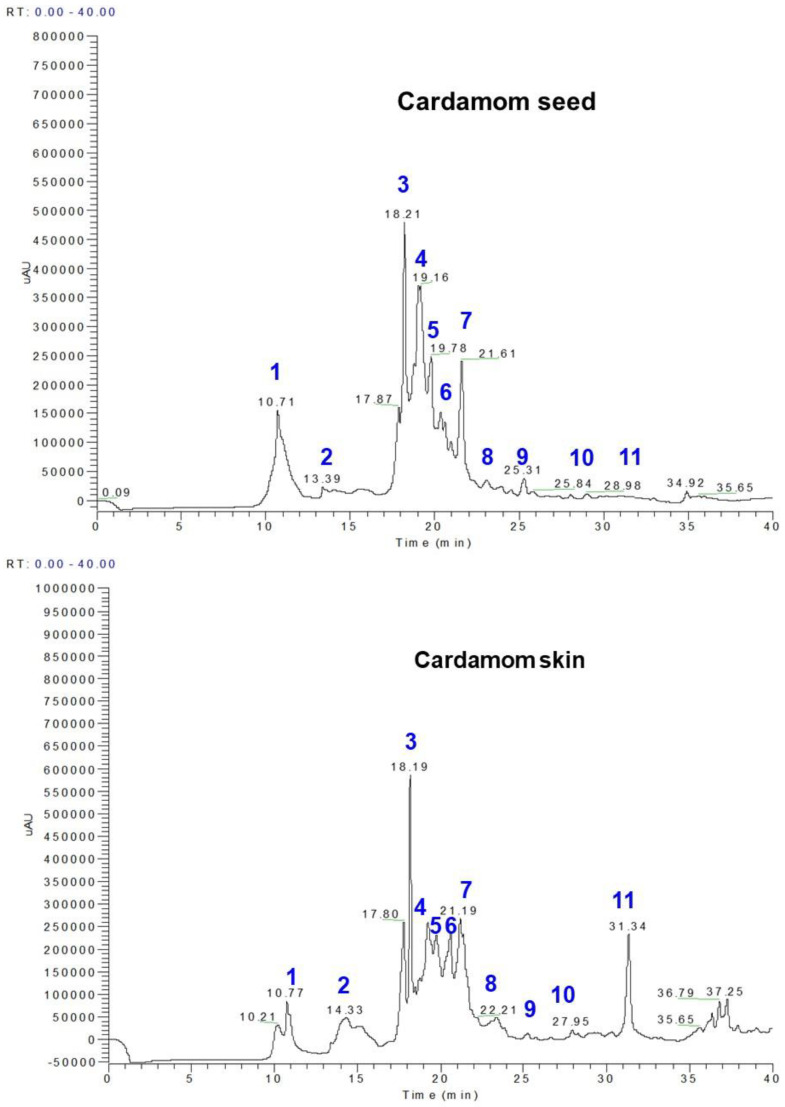

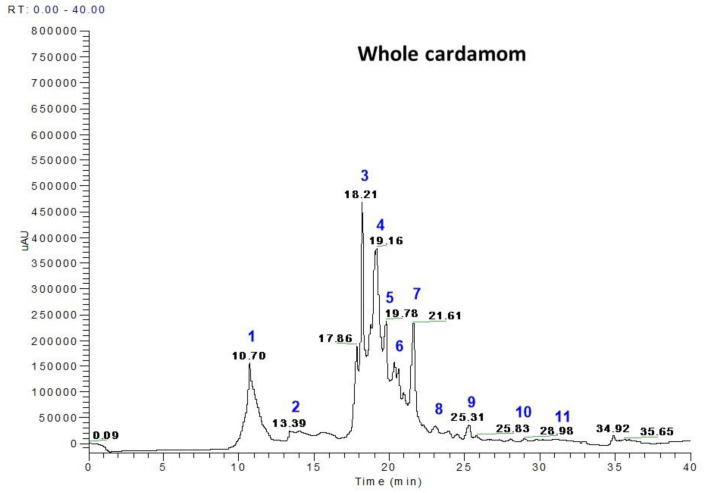
LC chromatograms of methanolic extracts of cardamom seed, cardamom skin, and whole cardamom. Individual peaks of phenolic compounds (peak numbers 1–11 in blue) were identified by LC-MS analysis and are reported in Table 2.

Herein, we identify the individual polyphenol compounds found in methanolic extracts of whole cardamom, skin, and seed samples by LC-MS analysis (Figure 1, Table 2). Peak **1** (10.70–10.78 min) showed a deprotonated ion [M-H]^−^ at *m*/*z* 153 in the negative mode ionization and yielded a major fragment at *m*/*z* 109 [M-H-44]^−^ due to the loss of the carboxylic acid group; hence, the peak was identified as protocatechuic acid. Protocatechuic acid was highest in the cardamom seed at 29.69 mg/100 g, followed by whole cardamom at 23.48 mg/100 g. Protocatechuic acid is the major human metabolite of cyanidin-glucosides [21]. Peak **2** (14.00–14.33 min) also gave a deprotonated ion [M-H]^−^ at *m*/*z* 153 and gave fragments at *m*/*z* 109 [M-H-44]^−^; hence, the compound was identified as gentisic acid. Gentisic acid was present in small amounts in the whole cardamom and seed and highest in cardamom skin at 3.87 mg/100 g. Gentisic acid is also present in the African tree Alchornea cordifolia, in wine [22], and as a hydroquinone, gentisic acid is readily oxidised and is used as an antioxidant excipient in some pharmaceutical preparations. In the laboratory, it is used as a sample matrix in matrix-assisted laser desorption/ionization (MALDI) [23] mass spectrometry and has been shown to conveniently detect peptides incorporating the boronic acid moiety by MALDI [24]. 

Peak **3** (18.20–18.21 min) gave [M-H]^−^ at *m*/*z* 179 due to the caffeic acid, and it gave major fragments at [M-H-18]^−^ *m*/*z* 161 and [M-H-44]^−^ *m*/*z* 135; hence, the compound was identified as caffeic acid and was highest in whole cardamom at 29.51 mg/100 g. Caffeic acid has a variety of potential pharmacological effects in in vitro studies and in animal models, and the inhibitory effect of caffeic acid on cancer cell proliferation by an oxidative mechanism in the human HT-1080 fibrosarcoma cell line has recently been established [25]. Caffeic acid is an antioxidant in vitro and in vivo and shows immunomodulatory and anti-inflammatory activity. Caffeic acid outperformed other antioxidants, reducing aflatoxin production by more than 95 percent [26]. Peak **4** (18.25–18.35 min) gave [M-H]^−^ at *m*/*z* 197 and yielded fragments at *m*/*z* 191 and *m*/*z* 173, and hence, the compound was identified as syringic acid. Syringic acid was abundant in both cardamom seed and whole cardamom at 36.43 mg/100 g and 34.11 mg/100 g, respectively. Syringic acid is also found in the açai palm [27] and wine. Its presence in the ancient Egyptian drink shedeh confirms that it was made out of grape, as syringic acid is released by the breakdown of the compound malvidin, also found in red wine. It is also found in vinegar [28]. Syringic acid can be enzymatically polymerized by laccase and peroxidase, for instance, inducing the polymerization of syringic acid to give a poly(1,4-phenylene oxide) bearing a carboxylic acid at one end and a phenolic hydroxyl group at the other [29]. 

Peak **5** (19.16–19.22 min) gave [M-H]^−^ at *m*/*z* 193 and yielded a fragment at *m*/*z* 179, and hence, this compound was identified as ferulic acid. Ferulic acid has been identified in Chinese medicinal herbs such as Angelica sinensis (female ginseng), Cimicifuga heracleifolia [30], and Ligusticum chuangxiong. It is also found in the tea brewed from the European centaury (Centaurium erythraea), a plant used as a medicinal herb in many parts of Europe [31]. Peak **6** (19.78–18.02 min) gave [M-H]^−^ at *m*/*z* 167 and gave fragments at *m*/*z* 135 and 121; hence, the compound was identified as vanillic acid. Vanillic acid is one of the main catechin metabolites found in humans after the consumption of green tea infusions. Peak **7** (20.8–21.19 min) gave [M-H]^−^ at *m*/*z* 353 and gave fragments at *m*/*z* 190 due to quinic acid and at 179 due to caffeic acid, suggesting a derivative of caffeoyl quinic acid; hence, the compound was identified as 5-O-caffeoyl quinic acid. The 5-caffeoyl quinic acid was almost double at 28.96 mg/100 g for whole cardamom as compared to the amount present in cardamom seed at 14.6 mg/100 g. It may have potential as a chemo-preventive dietary compound [32] and could be involved in the laxative effect observed in prunes [33].

Peak **8** (22.6–22.8 min) gave [M-H]^−^ at 163 and yielded fragments at *m*/*z* 143, 135, and 121; hence, the compound was identified as *p*-coumaric acid. *P*-coumaric acid is the precursor of 4-ethylphenol produced by the yeast Brettanomyces in wine. The yeast converts this to 4-vinylphenol via the enzyme cinnamate decarboxylase. Compound 4-vinylphenol is further reduced to 4-ethylphenol by the enzyme vinyl phenol reductase. Coumaric acid is sometimes added to microbiological media, enabling the positive identification of Brettanomyces by smell [34]. Peak **9** (23.08–24.8 min) gave [M-H]^−^ at *m*/*z* 397 and yielded fragments at [M-H-224]^−^ *m*/*z* 173 due to the loss of sinapic acid, and the fragment ion at *m*/*z* 191 was due to quinic acid. Hence, compound **9** was identified as sinapoyl quinic acid and was highest in whole cardamom at 5.79 mg/100 g. Sinapoyl quinic acid was in small amounts in the cardamom seed and skin extracts. Sinapoyl quinic acid was also detected in green Robusta coffee beans and has antioxidant properties [35]. Peak **10** (25.31–27.31 min) gave [M-H]^−^ at *m*/*z* 367 and yielded a fragment at [M-H-194] *m*/*z* 173 due to ferulic acid, and hence, the compound was identified as feruloyl quinic acid. Feruloyl quinic acid was present in very small quantities in both the whole cardamom and seed. Feruloyl quinic acid, along with caffeoyl quinic acids, are found abundantly in green coffee beans as a rich source of antioxidants [36]. 

Peak **11** (31.3–31.34 min) gave [M-H]^−^ at *m*/*z* 609 and gave fragments at [M-H-162]^−^ *m*/*z* 447 due to the loss of hexoside and [M-H-146] due to the loss of deoxy hexoside; hence, the compound was identified as rutin. Rutin levels were the highest in cardamom skin at 16.02 mg/100 g. Rutin and other dietary flavonols are under preliminary clinical research for their potential biological effects, such as in reducing post-thrombotic syndrome, venous insufficiency, and endothelial dysfunction, but there was no high-quality evidence for their safe and effective uses [37]. 

To our knowledge, this is the first detailed study on the polyphenol profiling of whole cardamom, skin, and seeds. Previous reports on cardamom phenolics show the identification of protocatechuic acid, gentisic acid, caffeic acid, vanillic acid, *p*-coumaric acid, and ferulic acid [38]. However, in the present study, we have found 11 phenolic compounds of which syringic acid, 5-O-caffeoylquinic acid, sinapoyl quinic acid, feruloyl quinic acid, and rutin are reported for the first time in whole cardamom, skin, and seeds. The total phenolic content was the sum of individual phenolics present in the seeds, skin, and whole cardamom extracts, giving ~124.5, 71.7, and 141.5 mg phenolics/100 g, respectively (Table 1). In general, syringic acid, protocatechuic acid, caffeic acid, and 5-O-caffeoylquinic acid were the major phenolics present in the seeds (~29, 23, 21, and 11%, respectively) and whole cardamom (~24, 16, 20, and 20%, respectively). On the other hand, caffeic acid and rutin were the major phenolics present in the skin (~26 and 22%, respectively) while vanillic acid, syringic acid, protocatechuic acid, and 5-O-caffeoylquinic acid were present in minor range of ~7–9% (Table 1).

### 3.2. Volatile Chemistry of Essential Oils from Whole Cardamom, Skin, and Seeds by GC-MS

**Figure 2:**
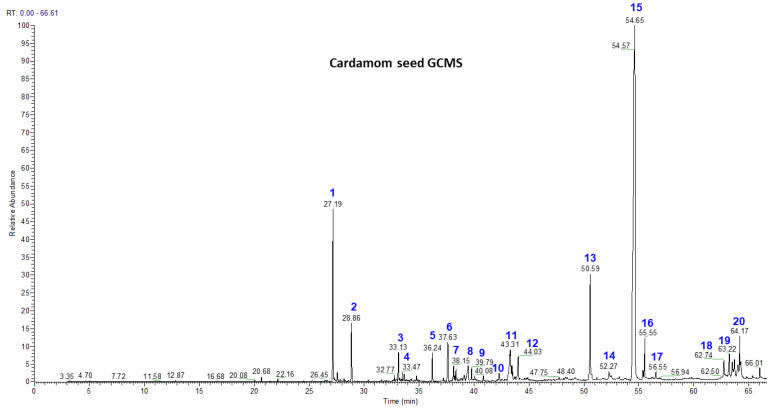

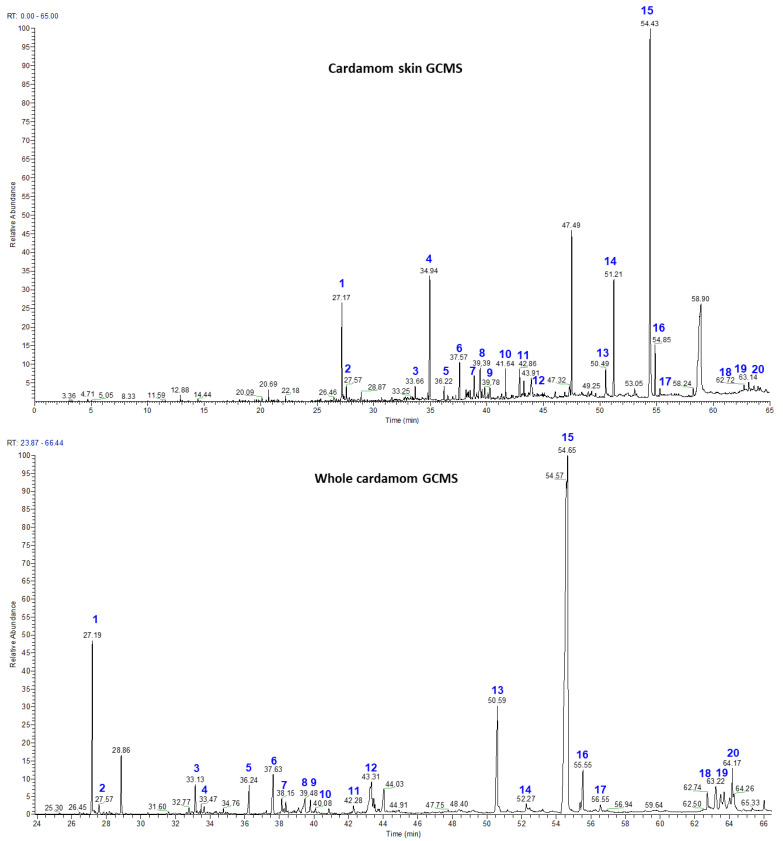
GC-MS analysis of cardamom seed, cardamom skin, and whole cardamom essential oils obtained by steam distillation. Individual peaks of volatile compounds (peak numbers 1–20 in blue) are reported in Table 3.

GC-MS analysis of the essential oil from whole cardamom, skin, and seeds gave the identification of 20 terpenes, among which costunolide (68.11%), ambrial (5.3%), and cis-α-terpineol (7.99%) were the major terpenes identified in cardamom seeds, while costunolide (29.8%), eicosane (5.53%), terpinen-4-ol (6.42%), and cis-α-terpineol (4.4%) were the major compounds found in cardamom skin (Figure 2, Table 3). Whole cardamom also contained major compounds such as costunolide (69.43%), ambrial (5.35%) and cis-α-terpineol (8.12%). Furthermore, α-acorenol, cubebol, isopathulenol, globulol, trans-farnesol, ambrial, eicosane, costunolide, coronarine, and erucylamide were some of the mono- and diterpenes which were not found in the literature in previous cardamom essential oil studies. In the present study, we also found fatty acid esters of palmitic and stearic acid as β-mono palmitin, β-mono stearin, and triterpene β-sitosterol. In the literature, the Indian variety of cardamom had its major compounds characterized as 1,8-cineole (38.7%), β-pinene (13.6%), α-terpineol (12.6%), spathulenol (8.3%), 4-terpineol (4.5%), germacrene-D (3.0%), α-pinene (2.8%), and β-selinene (2.7%) [39]. A recent study showed that cardamom essential oils possess greater antimicrobial activity than cumin with larger inhibition zones and lower minimum inhibitory concentrations for their antibacterial activities against Campylobacter jejuni and Campylobacter coli by using agar-well diffusion and broth microdilution methods, along with the mechanisms of antimicrobial action [40]. In general, the contents of essential oils in seed, skin, and whole cardamom were ~404, 422, and 416 mg/100 g, respectively. 

### 3.3. Colon and Macrophage Cell Viability, Reactive Oxygen Species (ROS), and Nitric Oxide (NO) Production 

**Figure 3:**
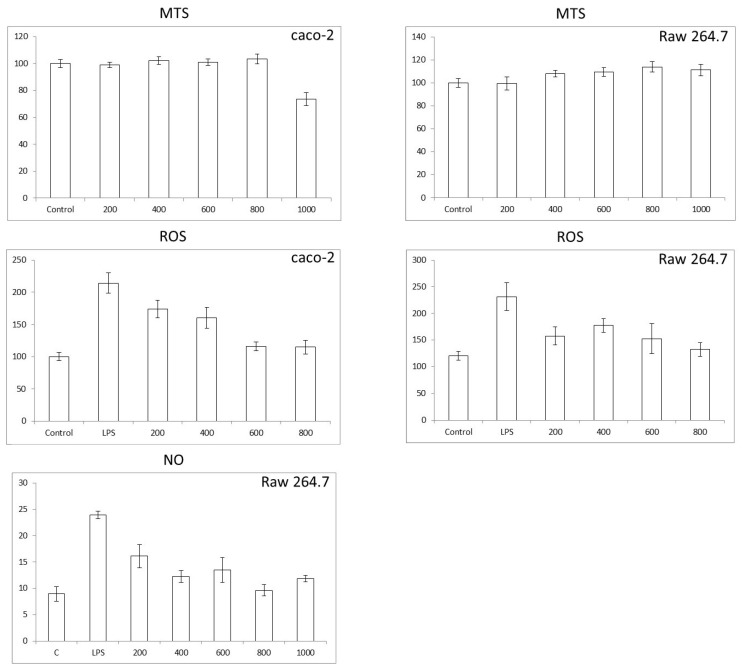
Cell viability (MTS test), reactive oxygen species (ROS), and nitric oxide (NO) production in colon and macrophage cells exposed to whole-cardamom methanolic extracts. Cell viability was assayed in colon Caco-2 cells (8  ×  10^3^ cells/well) and macrophage Raw 264.7 cells (5 ×  10^4^ cells/well) pretreated with 0–1000 µg/mL of whole-cardamom extract for 24 h. ROS production was assayed in Caco-2 cells (8  ×  10^3^ cells/well) and Raw 264.7 cells (5  ×  10^4^ cells/well), while NO was assayed in Raw 264.7 cells by pretreatment with 0–800 µg/mL of whole-cardamon extract for 5 h and then co-incubated for 19 h with 50 and 1 µg/mL LPS for Caco2 and Raw 264.7, respectively.

In the present study, the anti-inflammatory properties of whole-cardamom methanolic extracts were explored in macrophage and colon cells stimulated with an inflammatory bacterial lipopolysaccharide (LPS). Initial studies using the MTS test showed that whole-cardamom extracts in the range of 200–800 µg/mL (~1.9–7.9 µg phenolics/mL) did not affect cell viability in colon and macrophage cells; however, at 1000 µg/mL of extract (9.9 µg phenolics/mL) there was a ~30% decrease in cell viability in colon cells and detachment in macrophages (Figure 3). Colon and macrophages cells stimulated with LPS showed an increase in ROS by ~120% and 80%, respectively; however, ROS decreased in a dose-dependent manner when these cells were treated with cardamom extracts in the range of 200–800 µg/mL. The effective reduction of ROS to levels similar to the control values was observed in the range of 600–800 µg/mL (Figure 3). Furthermore, for macrophage cells challenged with LPS, there was an increase in NO levels by ~160%; however, these NO values decreased when cells were treated with cardamom extracts in the range of 200–800 µg/mL (Figure 3). These results indicate that cardamom polyphenols reduce oxidative stress induced by LPS and show the potential to reduce inflammation, as shown in the reduction of NO in macrophages. Further steps confirm the anti-inflammatory properties of whole-cardamom extracts by measuring the expression of pro-inflammatory genes.

### 3.4. Effects in Pro-Inflammatory Genes in Colon Cells

**Figure 4:**
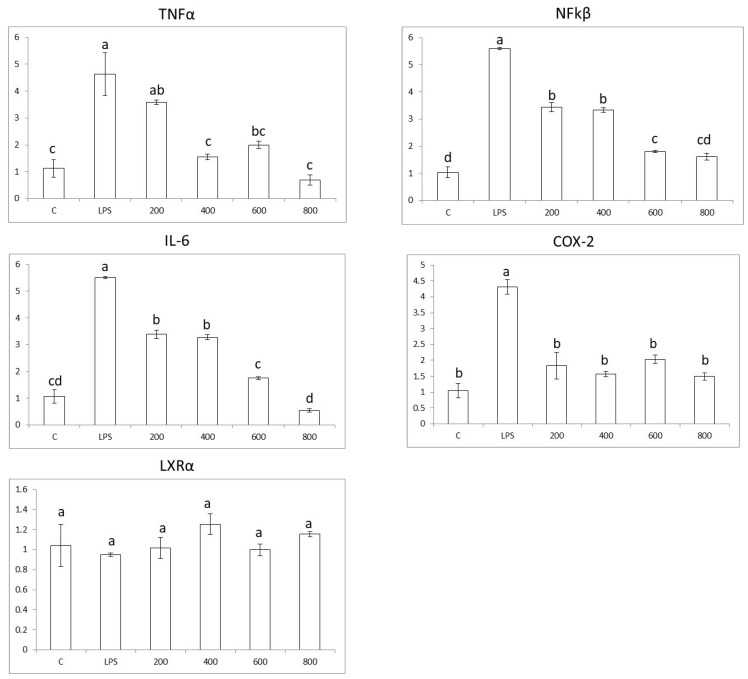
Anti-inflammatory properties of whole-cardamom methanolic extract in colon Caco-2 cells. Cardamom extract (200–800 µg/mL) effects on expression of pro-inflammatory genes (*NFkβ*, *TNFα*, *IL-6*, *COX-2*) and nuclear receptor *LXRα* were tested in colon cells (0.5 × 10^6^ cells/well) pre-incubated 5 h with cardamom extract and co-incubated afterwards 19 h with 50 µg/mL LPS. Cardamom extracts were dissolved in the growth medium including 0.5% DMSO and used as a control in all experiments. Different letters indicate significant differences among treatments at *p* < 0.05 by ANOVA and Tukey analysis.

Caco-2 colon cells, when exposed to LPS, show an increase in the expression of pro-inflammatory genes *NFkβ*, *TNFα*, *IL-6,* and *COX2* by ~5.5, 4.5, 5.5, and 4.5 times, respectively. Furthermore, the results show that whole-cardamom extracts in the range of 200–800 µg/mL have anti-inflammatory properties by lowering the expression of pro-inflammatory genes *NFkβ*, *TNFα*, *IL-6*, and *COX2* in a dose-dependent manner (Figure 4). The cardamom extract concentration required to reduce the expression of pro-inflammatory genes to control levels varied according to the specific gene; thus, for *NFkβ*, *TNFα*, *IL-6*, and *COX2*, the concentration values were 800, 400, 800, and 200 µg/mL, respectively.

**Figure 5:**
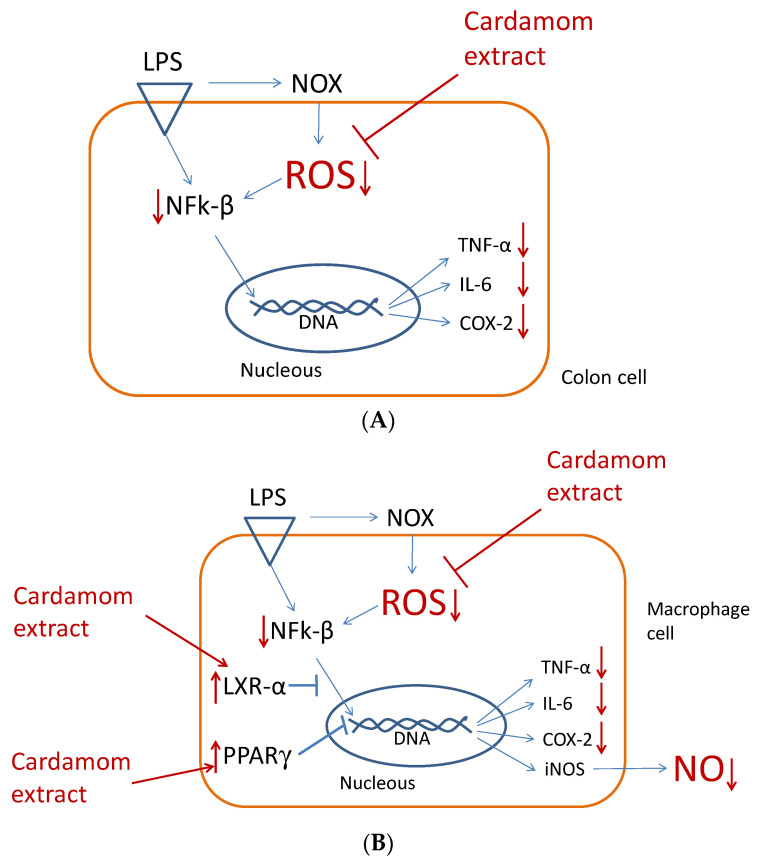
Anti-inflammatory dual mode of action of whole-cardamom methanolic extract in colon cells and macrophages. Bacteria lipopolysaccharide (LPS) binds to receptor TLR4 and triggers inflammation in colon and macrophage cells by activating NOX-enzyme-producing reactive oxygen species (ROS) that activate transcription factor NFk-β, which in turn upregulates pro-inflammatory genes. (**A**) In colon cells, cardamom extract decreases ROS and attenuates NFk-β, which in turn downregulate the pro-inflammatory genes of cytokines TNF-α and IL-6 and enzyme COX-2. (**B**) In macrophages, cardamom extracts show a dual mode of action by decreasing ROS and activating nuclear receptors LXR-α and PPARγ, which in turn attenuate NFk-β levels and downregulate the pro-inflammatory genes of cytokines TNF-α and IL-6 and enzymes COX-2 and iNOS, associated to NO production.

On the other hand, the expression nuclear receptor *LXRα* was not affected by LPS nor the cardamom extracts (Figure 4). Thus, the anti-inflammatory mode of action of cardamom extract on colon cells would be mainly through the reduction of oxidative stress (Figure 3). Accordingly, bacterial lipopolysaccharide (LPS) binds to the TLR4 receptor and triggers inflammation in colon cells by activating NADPH oxidase (NOX enzyme) and producing reactive oxygen species (ROS). The increased oxidative stress levels activate transcription factor NFk-β, which in turn upregulate pro-inflammatory cytokine genes *TNF-α*, *IL-6*, and enzyme *COX-2*. When cells are treated with whole-cardamom extract, there is a decrease in ROS levels likely due to scavenging properties, activating antioxidant enzyme activities, or both, which in turn attenuate NFk-β levels lowering the expression of pro-inflammatory genes in colon cells (Figure 5A).

### 3.5. Effects in Pro-Inflammatory Genes in Macrophages

**Figure 6:**
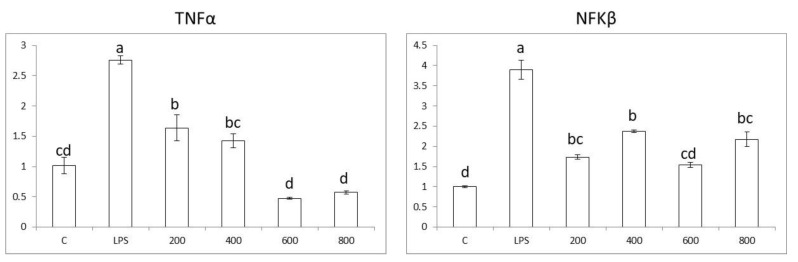

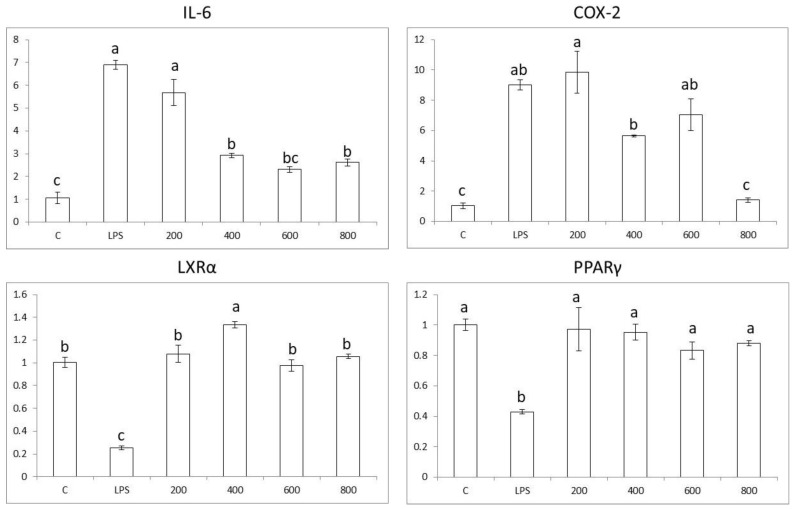
Anti-inflammatory properties of cardamom extract in Raw 264.7 macrophage cells. Cardamom extract (200–800 µg/mL) effects on expression of pro-inflammatory genes (*NFkβ*, *TNFα*, *IL-6*, *COX-2*) and nuclear receptors *LXRα* and *PPARγ* were tested in macrophage cells (0.5 × 10^6^ cells/well) pre-incubated 5 h with cardamom extract and co-incubated afterwards 19 h with 1 µg/mL LPS. Cardamom extracts were dissolved in the growth medium including 0.5% DMSO and used as a control in all experiments. Different letters indicate significant differences among treatments at *p* ≤ 0.05 by ANOVA and Tukey analyses.

Raw 264.7 macrophage cells, when exposed to LPS, show an increase in the expression of pro-inflammatory genes *NFkβ*, *TNFα*, *IL-6,* and *COX2* by ~4, 2.75, 7, and 9 times, respectively. When macrophage cells were treated with whole-cardamom extracts in the range of 200–800 µg/mL, there was an anti-inflammatory dose response in the expression of the pro-inflammatory genes *NFkβ*, *TNFα*, *IL-6,* and *COX2* (Figure 6) and a corresponding reduction in ROS and NO levels, as shown earlier (Figure 3). The cardamom extract concentration to reduce the expression of pro-inflammatory genes to control levels varied according to the specific gene; thus, for *NFkβ*, *TNFα*, *IL-6*, and *COX2*, the concentration values were 600, 400, 600, and 800 µg/mL, respectively. On the other hand, when cells were treated with LPS, there was a decrease in the genetic expression of nuclear receptors *LXRα* and *PPARγ* by ~80 and 60%, suggesting that the attenuation of nuclear receptors is involved in the activation of NFkβ during the inflammatory response in macrophage cells. Furthermore, when LPS-challenged macrophage cells were treated with whole-cardamom extracts, the gene expression of *LXRα* and *PPARγ* was restored to levels similar to the control at cardamon extract concentrations as low as 200 µg/mL (Figure 6).

These results suggest that the anti-inflammatory mode of action of cardamom extract on macrophage cells is mainly through the reduction of oxidative stress as well as by modulating nuclear receptors LXRα and PPARγ. Accordingly, bacterial lipopolysaccharide (LPS) binds to the TLR4 receptor and triggers inflammation in macrophage cells by activating NADPH oxidase (NOX enzyme) and producing reactive oxygen species (ROS) that partially activate the transcription factor NFk-β, which is also activated directly by TLR4. Increased levels of NFk-β are associated to the upregulation of pro-inflammatory cytokine genes *TNF-α* and *IL-6* and the gene expression of enzymes *COX-2* and *iNOS*, with the latter in turn producing NO. When cells are treated with the whole-cardamom extract, there is a decrease in ROS levels, likely due to scavenging properties of the cardamom polyphenols present in the extract or alternatively by enhancing the cell antioxidant system, or both. These polyphenols also increase LXRα and PPARγ levels which in turn attenuate NFk-β, resulting in decreased expressions of pro-inflammatory genes in macrophage cells and NO (Figure 5B). In this case, whole-cardamom extracts have an anti-inflammatory dual mode of action in macrophages by regulating ROS and the expression of nuclear receptors.

**Figure 7:**
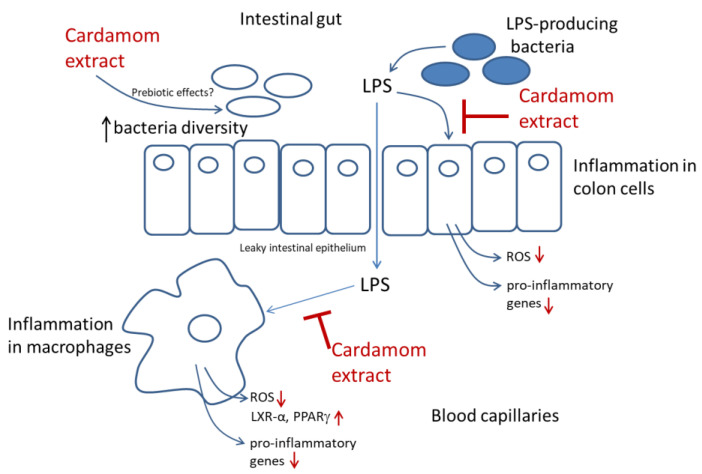
Hypothetical model of multifunctional effects of cardamom phenolic bioactives against low-grade inflammation. Cardamom may exert multifunctional effects against inflammation in three different fronts, including a possible prebiotic effect (to be confirmed) as well as by reducing LPS-induced inflammation in the intestinal epithelium due to diet-induced gut microbiota dysbiosis. In addition, cardamom may reduce LPS-induced inflammation in macrophages due to a leaky intestinal epithelium that allows LPS to reach the blood capillaries and induce inflammation in circulating macrophages and possibly initiate a low-grade inflammatory state.

Recently, an integrative model associating food, medicine, and chronic diseases was proposed, describing how low-grade systemic inflammation conduces a normal state of health to a pre-disease state, while a chronic inflammation state will sequentially conduce to a disease state [2]. Under this model, preventive and therapeutic strategies could be adopted based on controlling the inflammatory response. Accordingly, based on the present study, it is evident that the mode of action by which cardamom extract exerts its anti-inflammatory effects differs by cell type, and the bioactive compounds from cardamom extracts have multifunctional effects against inflammation working on different cells. Thus, when microbiota dysbiosis takes place due to an altered diet, LPS is produced, inducing inflammation in the intestinal epithelium. In addition, inflammation may induce a leaky intestinal epithelium, allowing LPS to reach the blood circulatory system and induce inflammation in circulating macrophages. Under this scenario, whole-cardamom extracts may reduce inflammation in colon cells by attenuating oxidative stress and may reduce inflammation in macrophages by a dual mode of action including reducing oxidative stress and activating nuclear receptors. Furthermore, future studies should explore the possible prebiotic effects of whole-cardamom extracts with the target of preventing dysbiosis and reducing LPS levels. Herein, we propose that the multifunctional effects of cardamom extracts in different fronts could be explored as preventive strategies to maintain a healthy gut by preventing low-grade inflammation as well as its transition to a chronic inflammatory scenario (Figure 7).

In general, whole cardamom contain high levels of phenolic and terpenoid compounds with contents of ~141.5 mg phenolics/100 g and 416 mg essential oil/100 g, respectively (Table 1, Section 3.4), giving a total bioactive content (phenolics + essential oil) of ~557.5 mg/100 g cardamom. However, due to the methanolic extraction procedure used in the present study, phenolics are the main group of compounds present in the cardamom extracts used in the cell studies with a negligible presence of terpenoids. The cardamom extract used in the range of 200–800 µg/mL is equivalent to 1.9–7.9 µg phenolics/mL. These cardamom phenolics are responsible for the anti-inflammatory and multifunctional effects observed in the present study. Nevertheless, further studies should be conducted with the terpenoid fraction (essential oil) to determine the anti-inflammatory potential of terpenoids since on weight basis, they represent ~74% of the total amount of bioactives present in whole cardamom. Previous studies showed that the terpenoid fraction in oregano [41] and jatropha [42] had anti-inflammatory properties. Furthermore, additional studies would be needed to determine which phenolic fractions in whole cardamom exerts higher bioactivity as shown previously in clitorea ternata, where flavonoid and anthocyanin fractions were reported to exert anti-inflammatory properties [43]. In addition, studies involving the microbiota metabolites of cardamom extracts are encouraged since recent studies with urolithins, which are microbial metabolites of ellagic acid, have shown they reduce lipid accumulation in adipocytes and reduce LPS-induced inflammation [44]. This generated information will lay the foundation for future in vivo studies using mouse models to confirm the anti-inflammatory properties and improvement of chronic diseases such as metabolic syndrome and define the dose of cardamom extract to be used in future clinical trials [45,46].

## 4. Conclusions

In the present study, chemical profiling of the polyphenolic and terpenoid compounds from whole cardamom, skin, and seeds showed 11 phenolics by LC-MS and 16 terpenoids by GC-MS, many of which are reported for the first time. A whole-cardamom methanolic extract showed anti-inflammatory properties in colon cells exposed to LPS by reducing oxidative stress and lowering the expression of pro-inflammatory genes *NFkβ*, *TNFα*, *IL-6,* and *COX2* while not affecting *LXRα*. The cardamom extract also showed anti-inflammatory properties in macrophages by reducing oxidative stress and enhancing the gene expression of nuclear receptors *LXRα* and *PPARγ*, which in turn lowered the expression of pro-inflammatory genes *NFkβ*, *TNFα*, *IL-6*, and *COX2* and reduced the levels of NO. Furthermore, cardamom extracts showed no toxicity effects on colon cells or macrophages in the range of 200–800 μg/mL. The whole-cardamom methanolic extracts contained high levels of phenolic compounds, which are likely responsible for the anti-inflammatory and multifunctional effects observed in the present study. This generated information suggests that cardamom can be used in diets for the prevention of chronic diseases associated with inflammation.

## Figures and Tables

**Table 1:** Primer sets for gene expression studies.

COX2	Fw-ACATCGATGTCATGGAACTG Rv-GGACACCCCTTCACATTATT
IL-6	Fw-TGACAACCACGGCCTTCCCT Rv-AGCCTCCGACTTGTGAAGTGGT
LXRα	Fw-AAGCCCTGCATGCCTACGT Rv-TGCAGACGCAGTGCAAACA
β-actin	Fw-CCCAGGCATTGCTGACAGG Rv-TGGAAGGTGGACAGTGAGGC
TNFα	Fw-ACTGGCAGAAGAGGCACTCC Rv-CGATCACCCCGAAGTTCA
PPARγ	Fw-GATGCACTGCCTATGAGCACTT Rv-AGAGGTCCACAGAGCTGATTCC
NFKβ	Fw-GGTGGAGGCATGTTCGGTA Rv-TGACCCCTGCGTTGGATT

**Table 2:** Identification of phenolic compounds from whole cardamom, skin, and seed by LC-MS analysis.

Peak No.	RT (min)	M-H	* MS Fragments	Identification	Seed (mg/100 g)	Skin (mg/100 g)	Whole Cardamom (mg/100 g)
1	10.70–10.78	153	**109**	Protocatechuic acid	29.69	6.09	23.48
2	14.00–14.33	153	**109**, 103	Gentisic acid	1.98	3.87	1.14
3	18.20–18.21	179	**161**, 135	Caffeic acid	26.23	18.73	29.51
4	18.25–18.35	197	191, 173	Syringic acid	36.43	6.69	34.11
5	19.16–19.22	193	**179**	Ferulic acid	6.68	4.11	8.51
6	19.78–18.02	167	**135**, 121	Vanillic acid	4.28	7.04	4.99
7	20.8–21.19	353	**190**, 179	5-O-caffeoylquinic acid	14.6	5.29	28.96
8	22.6–22.8	163	143, **135**, 121	*p*-coumaric acid	0.44	1.61	3.53
9	23.08–24.8	397	**191**, 173	Sinapoyl quinic acid	1.46	1.99	5.79
10	25.31–27.31	367	173, 161	Feruloyl quinic acid	0.054	0.27	0.97
11	31.3–31.34	609	447, **301**	Rutin	2.69	16.02	0.57

* Note: Bold numbers indicate the base peaks in MSn spectra.

**Table 3:** Identification of volatile compounds present in essential oils from whole cardamom, skin, and seed by GC-MS analysis.

Peak No.	Compound	Mass	Retention Time	Seed (% Area)	Skin (% Area)	Whole Cardamom (% Area)
1	Cis-α-Terpineol	154	27.17	7.99	4.4	8.12
2	Cis-β-Terpineol	154	27.57	1.43	0.84	1.55
3	Linalool	154	33.66	0.84	0.83	0.92
4	Terpinen-4-ol	154	34.94	0.23	6.42	0.33
5	α-Terpineol	154	36.22	0.92	1.31	0.98
6	Geraniol	154	37.57	1.05	2.2	1.22
7	Trans-Nerolidol	222	38.15	0.47	1.78	0.61
8	α-Acorenol	222	39.39	0.52	1.55	0.64
9	Cubebol	222	39.78	0.26	1.61	0.28
10	Isopathulenol	220	41.64	0.26	1.45	0.32
11	Globulol	222	42.24	0.75	1.64	0.79
12	Trans-Farnesol	222	43.91	0.62	1.18	0.73
13	Ambrial	234	50.49	5.3	1.52	5.35
14	Eicosane	282	51.21	0.22	5.53	0.28
15	Costunolide	232	54.43	68.11	29.8	69.43
16	Coronarin	300	54.85	1.12	2.73	1.25
17	β-Mono Palmitin	330	50.59	0.23	0.58	0.28
18	β-Mono Stearin	358	56.65	0.39	0.15	0.43
19	Erucylamide	337	62.71	0.41	0.23	0.48
20	β-Sitosterol	414	64.17	0.84	0.18	0.88

## Data Availability

Not applicable.

## Abbreviations

ROS	Reactive oxygen species
NO	Nitric oxide
LPS	Lipopolysaccharide
NFkβ	Nuclear factor kappa β
TNFα	Tumor necrosis factor alpha
IL-6	Interleukin 6
COX2	Cyclooxygenase 2
LXRα	Liver x receptor alpha
RNA	ribonucleic acid
PCR	Polymerase chain reaction
PPARγ	Peroxisome proliferator-activated receptor gamma
DMSO	Dimethyl sulfoxide
NOX	NADPH oxidase (nicotinamide adenine dinucleotide phosphate oxidase)
iNOS	Inducible nitric oxide synthase
